# Use of kernel-based Bayesian models to predict late osteolysis after hip replacement

**DOI:** 10.1098/rsif.2013.0678

**Published:** 2013-11-06

**Authors:** P. Aram, V. Kadirkamanathan, J. M. Wilkinson

**Affiliations:** 1 Department of Automatic Control and Systems Engineering, University of Sheffield, Sheffield, UK; 2 Academic Unit of Bone Metabolism, University of Sheffield, Sorby Wing, Northern General Hospital, Herries Road, Sheffield S5 7AU, UK

**Keywords:** kernel density estimation, Bayes theorem, biomaterials, osteolysis

## Abstract

We studied the relationship between osteolysis and polyethylene wear, age at surgery, body mass index and height in 463 subjects (180 osteolysis and 283 controls) after cemented Charnley total hip arthroplasty (THA), in order to develop a kernel-based Bayesian model to quantitate risk of osteolysis. Such tools may be integrated into decision-making algorithms to help personalize clinical decision-making. A predictive model was constructed, and the estimated posterior probability of the implant failure calculated. Annual wear provided the greatest discriminatory information. Age at surgery provided additional predictive information and was added to the model. Body mass index and height did not contain valuable discriminatory information over the range in which observations were densely sampled. The robustness and misclassification rate of the predictive model was evaluated by a five-times cross-validation method. This yielded a 70% correct predictive classification of subjects into osteolysis versus non-osteolysis groups at a mean of 11 years after THA. Finally, the data were divided into male and female subsets to further explore the relationship between wear rate, age at surgery and incidence of osteolysis. The correct classification rate using age and wear rate in the model was approximately 66% for males and 74% for females.

## 1. Introduction

Osteolysis, resulting in aseptic loosening, is the most common factor limiting the survival of modern total hip arthroplasty (THA). The pathogenesis of osteolysis is complex, with multiple factors contributing to its development [1]. Findings from several studies have suggested that polyethylene wear is the dominant factor in the development of osteolysis. The relationship between wear rate and the development of osteolysis has been characterized in a variety of statistical models. For example, the relationship between wear rate and osteolysis has been quantified using logistic regression analysis and expressing the results as odds of osteolysis per unit change in wear [2], and also using a population wear quintile-based approach to characterize the dose–response relationship between annual wear rate and osteolysis [3,4].

Measurement of polyethylene wear may be made clinically from plain radiographs, and several systems are available for this purpose [5–7]. Wear measurement made in the mid-term after THA have the potential to provide a tool for personalizing the need for later implant surveillance after THA. While these methods give useful information on the epidemiological association between wear and osteolysis, they do not provide risk data that would be directly clinically applicable to individual patients, and they also do not predict risk of osteolysis in the setting of other clinical risk factors, such as age and sex.

Intelligent decision support systems are commonly used in industry to give failure-time prediction based on multiple covariates that enables appropriate service intervals for mechanical parts, for example, the prediction of failures in aircraft engines [8]. This model, constructed by applying Bayes' theory and kernel density estimation, has been used extensively for pattern recognition in various fields, including in historical manuscript recognition [9], multiclass cancer classification [10] and *in situ* hybridization signal classification [11]. In this study, we aimed to explore the potential application of this tool to compute the probability of implant failure using multiple risk factors. We aimed to use predictor variables that could easily be obtained in the clinical setting to construct the model such that a simple but multivariate-based estimate of risk could be calculated.

## 2. Material and methods

### 2.1. Subjects

The subject data used in this analysis were collected as part of a study examining patient risk factors for osteolysis [12]. The study was approved by the local ethics committee, and all subjects provided written, informed consent prior to participation. Subjects were recruited between February 2000 and April 2006 and included Caucasian men and women who had previously undergone THA for idiopathic osteoarthritis of the hip between the years 1971 and 1998. The exclusion criteria for this study are detailed elsewhere [12]. The anonymized supporting data are accessible through Sheffield Musculoskeletal Biobank via request to the senior author.

All subjects received a cemented monobloc Charnley femoral component with a 22 mm diameter femoral head, and a cemented Charnley polyethylene acetabular component. The osteolysis group comprised 180 subjects who have subsequently undergone revision surgery for osteolysis or aseptic loosening. Loosening of the femoral component was defined according to the criteria of Harris & McGann [13], and loosening of the acetabular component was defined according to the criteria of Harris & Penenberg [14]. The control group comprised 283 subjects with well-functioning implants at a mean of 12 years (s.d. = 4) after surgery, with no radiological evidence of loosening (table 1). Annual linear wear rate was measured on digitized plain radiographs of the pelvis using a uniradiographic technique with EBRA-Digital software (v. 2000, University of Innsbruck, Austria). Use of this technique and its precision in our institution is detailed elsewhere [15].

**Table 1.** Characteristics of study subjects. Plus–minus figures are mean ± s.d.

patient characteristics	Charnley THA control (*n* = 283)	Charnley THA osteolysis (*n* = 180)
sex (male-female)	132-151	106-74
height (m)	1.63 ± 0.09	1.67 ± 0.09
weight (kg)	76.0 ± 15.3	79.1 ± 15.0
body mass index (kg m^–2^)	28.4 ± 5.0	28.4 ± 4.6
implant survival time (years)	11.9 ± 4.2	10.2 ± 4.7
age at surgery (years)	64.1 ± 8.2	59.5 ± 8.8
total wear (mm)	1.022 ± 0.957	1.411 ± 0.955

### 2.2. Model development

#### 2.2.1. Background theory and development of kernel density estimators

Prediction of the development of osteolysis can be viewed as a classification problem, as the outcome is a binary variable. The classification task can be considered as assigning probabilities to each class *C_i_*, {*i* = 1, … *M*} among *M* classes given some observation 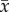, expressed as 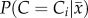. Using Bayes' theorem, we have2.1
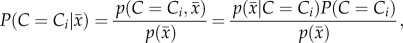
where *P*(*C* = *C_i_*) is the prior probability of class *i*, 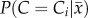 is the posterior probability of class *i* given the observation 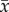, and 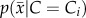 is the likelihood function or conditional probability density of observation 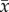 given the class *C_i_*. For an *M*-class classification problem, we have [16]2.2
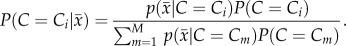
To apply equation (2.2), for each class, we need prior probability and the probability density function of data given class membership. Assuming large enough data samples, an estimate of the prior probability can be obtained from the relative frequency of occurrence of data with known class. We calculate conditional probability density, using a non-parametric kernel estimate [17]2.3
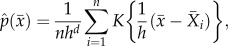
*K*(.) denotes the kernel function (satisfying appropriate constraints, e.g. 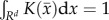, 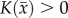, etc.) and *h* is the window width, also called the smoothing parameter or bandwidth. *n* is the number of observations 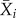 with the dimension *d*. The range of 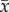 depends on the sampled data. The kernel estimator is a sum of kernel functions placed at the data points. Normally, *K*(.) is chosen to be a radially symmetric probability density function, such as the standard multivariate normal density function2.4
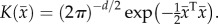
The effective use of kernel density model is subject to an appropriate choice of the window width parameter. This can be seen as a trade-off between the bias and the variance in the estimates. A very small value of *h* causes random variations to appear in density estimates, while choosing a large value for *h* may eliminate the important characteristics (bimodality for instance) of the underlying distribution.

There are several approaches to the estimation of the window width parameter, *h* [18]. For example in the one-dimensional case, the optimal value of *h* under the assumption that data are distributed normally is given by *h* = (4/3*n*)^1/5^ *σ*, where *σ* is the standard deviation of the data. However, data such as wear rate are typically not distributed normally. In order to deal with asymmetric, long-tailed distributions and outliers, a robust estimate of *σ* such as the median absolute deviation estimator is more desirable [19]. This leads to the choice2.5
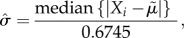
where 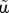 denotes the median of the sample.

It should be noted that the appropriate method for choosing the window width depends on the application and the nature of the dataset. In some applications, visual tuning of *h* based on prior knowledge about the underlying population can be sufficient while in others more sophisticated automatic methods such as least-squares cross-validation [20] or smoothed bootstrap [21] are required.

#### 2.2.2. Univariate analysis and construction of a bivariate model

The wear rate data were distributed in a lognormal fashion with a long right-handed tail and were log transformed to normalize the distribution before its inclusion in the model. A kernel density estimator with fixed window width was used to construct the class conditional density using the wear rate data. When the smoothing parameter of *h* = 0.3 based on the robust estimate of the standard deviation (equation (2.5)) was used, the density function was not sufficiently smooth. The window width of the smoothing parameter was increased to 0.7 to obtain a more smooth estimation. The same window width was also used for age at surgery, body mass index and height, to estimate class conditional densities for control and revision subjects in the training set. The prior probability of osteolysis was obtained from the relative frequency of occurrence of data with known class, *P*(*C* = *C*_0_) = 0.612 and *P*(*C* = *C*_1_) = 0.388, where *C*_0_ denotes control group and *C_1_* denotes revision group. Classification was made using Bayesian decision theory using prior probabilities and estimated class conditional densities for the training set for all the features separately. For the bivariate case, each of the other features (age, height and body mass index) was paired separately with annual wear rate.

#### 2.2.3. Sex-specific model and cross-validation

In order to test the validity of the model in terms of its predictive value, we applied the *k*-fold cross-validation [22]. This allowed all available subject data to be used for training the model, and allowed the misclassification rate of the model to be calculated. The data are divided into *k* random subsets of approximately equal size. The model is then trained *k* times using data from *k* – 1 of the subsets. Each time a single subset was left out to serve temporally as an independent test sample for evaluating the desired performance criterion. A good estimate of the classification performance is given by the average performance over the *k* independent tests. In this study, the value of *k* was taken as 5. Adopting the five-fold cross-validation procedure, subjects were randomly divided into five distinct segments in order to examine the robustness of the model (table 2). Each subgroup was randomly made up of 36 revision and approximately 57 control subjects. In the multivariable case, observations were transformed to have zero-mean and unit-variance data so that they were dimensionless and had the same spread and similar range, allowing a standard kernel to be used. In order to deal with long-tailed annual wear distribution the natural logarithm of annual wear was used to normalize the data distribution. Then the five test sample estimates were averaged to obtain the estimate of misclassification percentage.

**Table 2.** Mean values of the five-fold cross-validation dataset: control group (top); osteolysis group (bottom).

set	total wear (mm)	age at surgery (years)	height (m)	weight (kg)	body mass index (kg m^–2^)	osteolysis-free survival time (years)
1	0.970	63.5	1.62	73.2	28.0	11.5
2	1.181	64.9	1.64	75.7	28.2	12.1
3	0.872	65.4	1.62	73.9	28.2	11.5
4	1.011	64.3	1.64	76.5	28.4	12.3
5	1.082	62.3	1.66	80.7	29.4	12.2
mean	1.023	64.1	1.63	76.0	28.4	11.9
1	1.308	62.8	1.69	80.4	27.9	9.2
2	1.404	58.5	1.66	79.6	28.9	10.0
3	1.487	60.8	1.66	76.0	27.3	9.4
4	1.331	57.6	1.65	79.3	29.2	11.2
5	1.523	57.5	1.68	90.1	28.5	11.2
mean	1.411	59.5	1.67	79.1	28.4	10.2

Finally, male and female subjects were studied separately to further investigate the sex-specific relationship between wear rate, age at surgery and incidence of osteolysis, and to calculate the misclassification rate for males and females independently.

#### 2.2.4. Sensitivity analysis

As mentioned earlier, the window width parameter governs the smoothness of the density estimation, which consequently affects the resulting posterior probability obtained by equation (2.2). To study the sensitivity of the classifier output to the window width, different values of *h* over the range of 0.1 and 10 with step size 0.1 were used to compute the class conditional densities. The five-fold cross-validation method was performed over 100 permutations of data for each classifier. The misclassification rate was then averaged over 100 permutations. This was performed for the bivariate model using the entire dataset and also male and female groups separately.

Different runs of five-fold cross-validation provide different misclassification rates due to the effect of random variation in selecting each subset. By rerunning the cross-validation method several times, a more accurate misclassification rate can be calculated [23], to better characterize the sensitivity of the model to the window width variations.

## 3. Results

### 3.1. Univariate analysis and construction of a multivariate model

**Figure 1.**
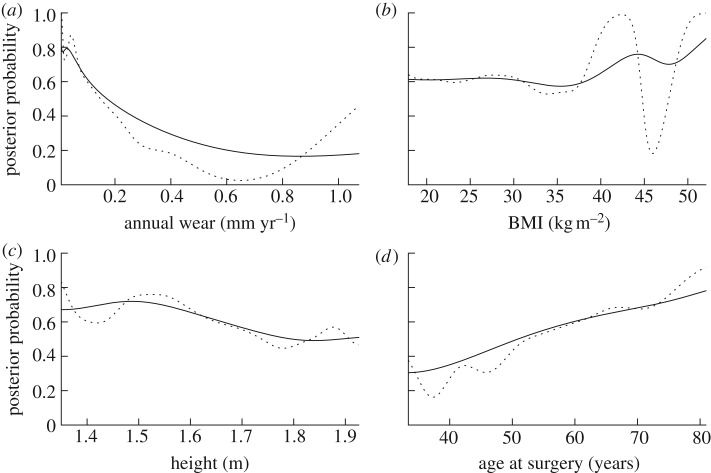
The posterior probability of implant survival at 11 years based on (*a*) log implant wear rate, (*b*) BMI, (*c*) height and (*d*) age at surgery. The posterior probabilities for *h* = 0.3 and *h* = 0.7 are shown by dotted and solid lines, respectively. Wear data have been back transformed to allow interpretation in the setting of clinical wear measurements.

**Figure 2.**
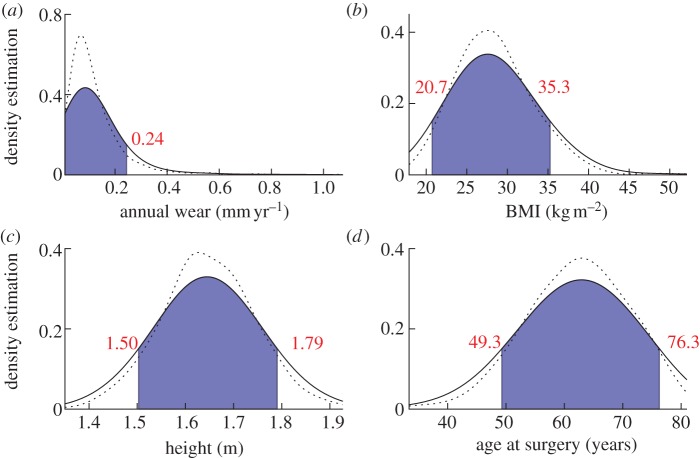
Density estimation of the whole dataset indicating regions where data are most densely sampled; (*a*) log implant wear rate, (*b*) BMI, (*c*) height and (*d*) age at surgery. The density estimations for *h* = 0.3 and *h* = 0.7 are shown by dotted and solid lines, respectively. The blue shaded areas show regions where data is densely sampled with the range indicated in each subplots. Wear data have been back transformed to allow interpretation in the setting of clinical wear measurements.

The posterior probabilities of the univariate case for annual wear using normalized log-transformed wear data, BMI, height and age at surgery with linear normalization are shown in figure 1. The smoothing parameter in this case was *h* = 0.7. We can infer that the probability of a patient with annual wear 0.2 (mm/year) being in control group is 0.45 (figure 1*a*). The range of posterior probability variation for BMI is between 0.57 and 0.61 over the interval within which our data are concentrated (BMI values of 20.7–35.3, figure 1*b*), therefore this parameter in isolation does not contain valuable discriminatory information. The same conclusion can be made for height (values in the range 1.50–1.79; figure 1*c*). However, age at surgery contained more information over the range in which observations are densely sampled (49.3–76.3, figure 1*d*). The density estimation of each variable using the whole dataset is also calculated showing regions where densely sampled data are available (figure 2*a*–*d*).

**Figure 3.**
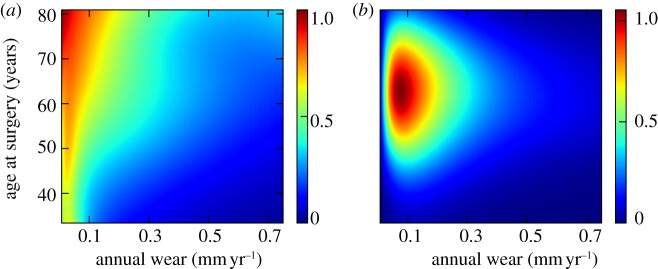
(*a*) Prediction of implant survival based on age at surgery and log implant wear rate (colour indicates posterior probability of implant survival at 11 years); (*b*) joint density estimation indicates regions where data are most densely sampled. Wear data have been back transformed to allow interpretation in the setting of clinical wear measurements.

A predictive model based on annual wear rate was chosen as the most informative univariate model. To develop the model further into a multivariable model, we included each remaining feature. Linear normalized age at surgery and normalized log-transformed annual wear were used. Annual wear rate with age at surgery had the highest discriminatory information among all possible bivariate models over the ranges in which observations were densely sampled. The risk of osteolysis decreased as age at surgery increased for a given wear rate (figure 3*a*) over the area where the data were densely distributed (figure 3*b*).

### 3.2. Sex-specific model and cross-validation

**Figure 4.**
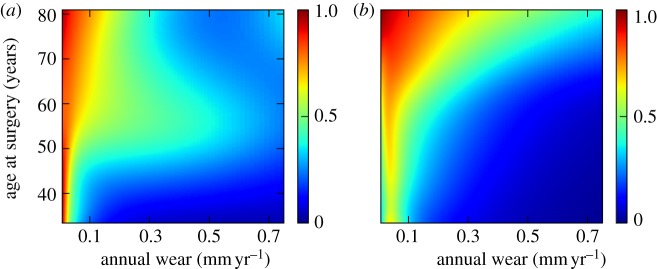
Sex-specific prediction of implant survival based on age at surgery and implant wear rate; (*a*) male group and (*b*) female group. Wear data have been back transformed to allow interpretation in the setting of clinical wear measurements.

**Figure 5.**
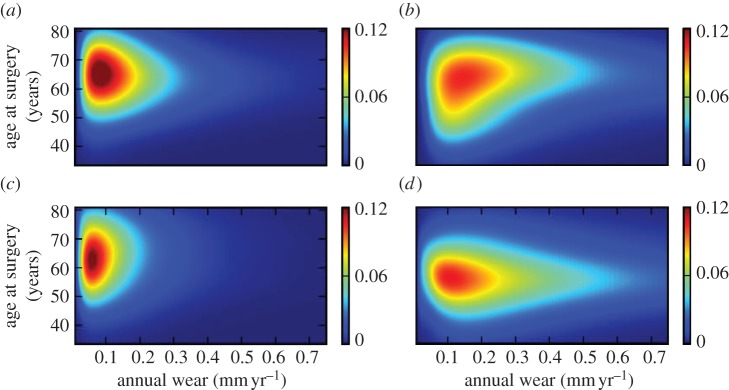
Sex-specific density estimation based on age at surgery and implant wear rate; (*a*) male control group, (*b*) male revision group, (*c*) female control group and (*d*) female revision group. Wear data have been back transformed to allow interpretation in the setting of clinical wear measurements.

The characteristics of the subjects randomly allocated to each of the five cross-validation subsets were similar (table 2). The mean misclassification rate calculated using this cross-validation method was 30.6% (range 25.8–34.7). The predictive value of the model of implant survival based on age at surgery and implant wear for male and female subjects analysed independently demonstrated better predictive value of the model for females versus males. The mean misclassification percentage of five-fold cross-validation within the overall dataset was 31% (s.d. 3; range 26–35). The misclassification rate for men was 34% (6; 26–40), and for females was 26% (3; 23–31: *p* = 0.04). The prediction of implant survival based on age at surgery and annual wear rate for male and female subjects are shown in figure 4*a*,*b*, respectively. The sex-specific probability density estimation for both control and revision groups are also shown in figure 5. The inferior performance for males can be attributed to the long-tailed density of the control group (figure 5*a*) compared to its female counterpart (figure 5*c*). There are also fewer data points with high wear rate in the male revision group (figure 5*b*) compared with the female revision group (figure 5*d*). This perhaps resulted in a better training of the model over the region with high annual wear rate where only female subjects were used.

### 3.3. Sensitivity analysis

**Figure 6.**
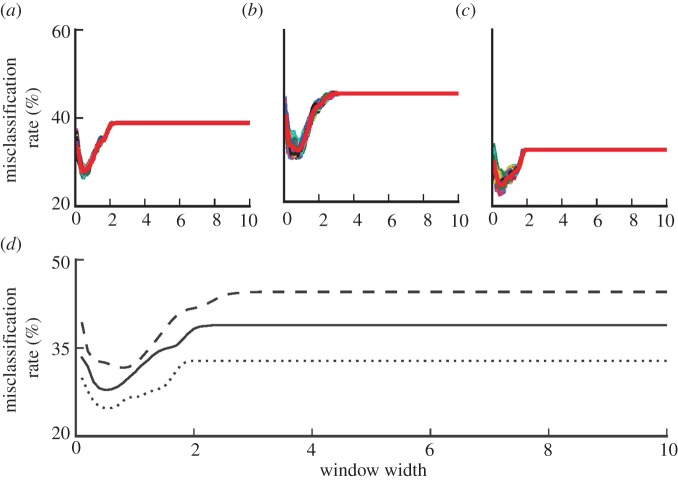
The effect of the window width variations on the misclassification percentage; (*a*) misclassification rate of bivariate model based on age at surgery and log implant wear rate over 100 permutations of the whole dataset, (*b*) male group, (*c*) female group, (*d*) the comparison of the averaged (over 100 permutations) of the misclassification rates for (*a*–*c*). Coloured lines show the misclassifications of each permutation. Thick red lines show the average misclassification rate over 100 trials. In the lower panel, misclassification of the whole dataset (solid line), male group (dashed line) and female group (dotted line) are re-plotted for better comparison.

The mean misclassification rates using five-fold cross-validation method for 100 permutations of the data and different values of *h* were computed. The results for the overall dataset, male and female groups are shown in figure 6*a*–*c*, respectively. The average misclassification rates are also re-plotted in figure 6*d* for better visualization. The model provides a better performance for the female group over the entire range of the window width. The average misclassification rate for data including both male and female sexes was between 28 and 39%, for male was 31 and 44% and for female was 25 and 33%. The high misclassification rate for small *h* is due to the high variations of the class conditional density estimates. For large values of *h*, the posterior probability obtained based on kernel density estimation approaches prior probability of the control group, corresponding to the constant sections of the misclassification rates in figure 5. The convergence of the posterior probability to the prior holds irrespective of the problem and the dataset and therefore does not carry any discriminatory information [24]. The best performance for the overall dataset and female group corresponded to *h* = 0.5 and for male group corresponded to *h* = 0.8. In the previous analyses, a value between these two, i.e. *h* = 0.7 was chosen.

## 4. Discussion

In this study, we examined the potential role of a kernel-based Bayesian model in the prediction of osteolysis in the late period after cemented THA. We found that mean annual wear rate was the most predictive variable for osteolysis, followed by age at THA surgery. This model, based on Bayes' theory, gave a correct classification rate for osteolysis of approximately 70%, using five-fold cross-validation to examine the accuracy of the model. When the data were divided into male and female subsets, the correct classification rate for female was 74% versus 66% for males.

The appropriate choice of the window width is crucial in constructing the classifier. There are various methods suggested in the literature to calculate the window width parameter [18], which can be easily incorporated to the proposed method in this work. One simple technique is to determine the window width based on the sample standard deviation or its alternative robust estimates [19]. This works well if the observation points are normally distributed. Other commonly used methods are based on cross-validation or bootstrap techniques, which could impose high computational costs when applied on very large datasets. The bootstrap based techniques shown to be superior to the cross-validation based methods, however, are computationally more expensive [21]. It should be noted that these methods might have different performances for different datasets. For the dataset used in this work, our sensitivity analysis shows values that prevent noisy and over smoothed density estimations can provide approximately similar misclassification rates.

The finding that mean annual wear rate and age at surgery were predictive factors for osteolysis is consistent with the findings of several other studies highlighting these patient factors as risk factors for osteolysis [2,25–27]. While these previous studies have quantitated the contribution of these variables to the risk of osteolysis, these analyses have been conducted and the data expressed as univariate survivorship analyses and proportional hazards. However, such analyses cannot be easily applied to provide risk prediction in the clinical setting, as the predictive risk factors are independent variables, and the contribution to each in the overall risk needs to be incorporated to allow personalized prediction of prosthesis survival. The statistical approach taken here contrasts with previous studies in that we have aimed to use the demographic patient data to construct a multivariate model for predicting osteolysis that might be applicable to estimate risk for individual patients in clinical practice. We have shown that the likelihood of osteolysis at a mean of 11 years after surgery may be calculated from patient-specific factors, such as age at surgery, wear rate and sex. The cross-validation data suggest that the error rate with this method is approximately 30%.

We have used a uniradiographic method for calculating mean annual wear rate. This method assumes that wear rate is fairly constant over time. Previous longitudinal studies of wear rates suggest that this assumption is valid, provided that wear rate is calculated from data collected after the initial run in wear period that lasts 1–2 years. This model has been derived from a retrospective dataset, however, and needs to be confirmed prospectively to evaluate its robustness in clinical practice. Furthermore, the derived estimates are likely to be prosthesis-specific, as the relationship between linear and volumetric wear rates is dependent on the diameter of the bearing articulation, and may differ between cemented and cementless implants. Collection of prospective datasets to validate and inform the refinement of this predictive model might also include other potentially relevant variables, such as patient activity levels that also may impact on prosthesis survival modelling.

The clinical dataset used to develop this statistical model was based on the Charnley monobloc hip replacement that uses a 22 mm head and a metal on conventional polyethylene bearing. The estimates of the precise contribution of individual predictor variables, and the amount of total variability in the outcome variable generated here is thus only directly applicable to the Charnley 22 mm prosthesis in our specific population. However, in this paper, we aimed to demonstrate the broader proof that this Bayesian statistical approach can be applied to generate an accurate multivariate prosthesis survivorship tool that would have utility in personalized clinical prediction. We chose the Charnley prosthesis as the exemplar for this proof of principle as it is a benchmark prosthesis that has well-characterized survivorship behaviour.

We have included subjects with both femoral and pelvic osteolysis in the dataset from which the model was generated. In this analysis, we did not divide the datasets into femoral versus pelvic osteolysis, as the patient-relevant endpoint for surveillance purposes is to predict the need for a revision surgery episode. Finally, this type of model does not incorporate individual patient's biological response to wear particulate debris as a predictor variable, which may also be an important consideration in the development of osteolysis after THA [28].

In summary, predictive models adapted from the industrial setting may provide a useful additional strategy in identifying patients at risk of osteolysis and for stratifying clinical follow up according to risk. This Bayesian model performs well where modelling data are available for densely sampled regions within the model.
